# Adult Tea Green Leafhoppers, *Empoasca onukii* (Matsuda), Change Behaviors under Varying Light Conditions

**DOI:** 10.1371/journal.pone.0168439

**Published:** 2017-01-19

**Authors:** Longqing Shi, Liette Vasseur, Huoshui Huang, Zhaohua Zeng, Guiping Hu, Xin Liu, Minsheng You

**Affiliations:** 1 Institute of Applied Ecology and Research Centre for Biodiversity and Eco-Safety, Fujian Agriculture and Forestry University, Fuzhou, China; 2 Fujian-Taiwan Joint Innovation Centre for Ecological Control of Crop Pests, Fujian Agriculture and Forestry University, Fuzhou, China; 3 Key Laboratory of Integrated Pest Management of Fujian and Taiwan, China Ministry of Agriculture, Fuzhou, China; 4 Department of Biological Sciences, Brock University, 1812 Sir Isaac Brock Way, St. Catharines, ON L2S 3A1, Canada; 5 Quanzhou Entry-exit Inspection and Quarantine Bureau of People's Republic of China, Quanzhou, China; 6 Institute of Plant Protection, Fujian Academy of Agricultural Sciences, Fuzhou, China; 7 Jiangxi Serculture and Tea Research Institute, Nanchang County, Nanchang City, Jiangxi Province, People's Republic of China; University of Idaho, UNITED STATES

## Abstract

Insect behaviors are often influenced by light conditions including photoperiod, light intensity, and wavelength. Understanding pest insect responses to changing light conditions may help with developing alternative strategies for pest control. Little is known about the behavioral responses of leafhoppers (Hemiptera: Cicadellidae) to light conditions. The behavior of the tea green leafhopper, *Empoasca onukii* Matsuda, was examined when exposed to different light photoperiods or wavelengths. Observations included the frequency of locomotion and cleaning activities, and the duration of time spent searching. The results suggested that under normal photoperiod both female and male adults were generally more active in darkness (i.e., at night) than in light. In continuous darkness (DD), the locomotion and cleaning events in Period 1 (7:00–19:00) were significantly increased, when compared to the leafhoppers under normal photoperiod (LD). Leafhoppers, especially females, changed their behavioral patterns to a two day cycle under DD. Under continuous illumination (continuous quartz lamp light, yellow light at night, and green light at night), the activities of locomotion, cleaning, and searching were significantly suppressed during the night (19:00–7:00) and locomotion activities of both females and males were significantly increased during the day (7:00–19:00), suggesting a shift in circadian rhythm. Our work suggests that changes in light conditions, including photoperiod and wavelength, can influence behavioral activities of leafhoppers, potentially affecting other life history traits such as reproduction and development, and may serve as a method for leafhopper behavioral control.

## Introduction

Insects can be highly influenced by light conditions including photoperiod, light intensity, and wavelength [1, 2, 3]. Apart from the widely known diapause phenomenon [4, 5], photoperiod influences various behaviors such as egg hatching, flight, locomotion, feeding, courtship, and mating [6, 7]. For instance, walking, foraging, and oviposition of *Frankliniella occidentalis* Pergande are increased with increasing time of illumination but reduced in continuous darkness [8]. Many *Drosophila* species and the tephritid fly *Anastrepha ludens* have their mating success reduced when exposed to continuous darkness [9, 10]. The influence of wavelength varies among insect species. Insects tend to be most sensitive to blue or blue–green regions of the spectrum (400–550 nm) [3]. Short wavelengths stimulate the flight and inhibit landing of the whitefly *Trialeurodes vaporariorum*, while long wavelengths stimulate landing and inhibit flight [11]. Reproduction and development of ladybird beetles, ants, and honeybees are negatively affected by red light [12]. Meanwhile, green light has been found to reduce reproduction in *Propylea japonica* [13]. In Japan, as a pest management approach, yellow and green lights have been used at night to suppress behavioral activities of nocturnal moths [6].

The tea green leafhopper, *Empoasca onukii* Matsuda (Hemiptera: Cicadellidae), is one of the main insect pests in tea plantations in Asia [14, 15]. There are five nymphal instars. Courtship and mating occur 4–5 days after emergence. Usually, the adult females are larger than the males and live longer [16]. All stages can cause serious damage to tea plants. Nymphs and adults suck sap from young tea shoots and leaves and the eggs are laid into the branches, causing damage called hopperburn [17]. In China, *E*. *onukii* has 9–17 generations per year depending on regional climatic conditions. The most serious outbreaks normally occur in June and September, resulting in loss of tea production [16, 18]. So far, little is known about the behavior of the tea green leafhopper, especially the influence of changing light conditions. Variation in photoperiod can lead to changes in circadian rhythm affecting behavior and potentially fitness [18]. In adult tea green leafhoppers, three major behavioral activities can be observed: cleaning, locomotion, and searching. Cleaning is an essential behavior that enhances hydrophobicity through the coating of their integuments with brochosomes, thus protecting the adults from being washed off from leaves by water [19, 20]. Locomotion activities, such as walking, jumping and flying, are considered important elements of survival as they are usually related to avoiding danger (for instance, enemies), foraging, finding places to stand and social interactions [21, 22]. Searching is crucial especially during courtship as the male leafhopper continually flies among plants until it finds a mate. When in proximity of a female, the male initiates its courtship behavior using vibration signals produced from the abdomen. A receptive female will then respond to the vibration signals and allows the male to move closer to her. In this period of time, females barely move [23, 24, 25]. Investigating the adaptive behavioral responses of leafhoppers to the influence of light can help better understand its adaptive capacity to respond to environmental changes and gradually develop alternative insect control methods.

In this study, we investigated the behavioral responses of *E*. *onukii* under different indoor light environments. Adult females and males were exposed to different photoperiods and light wavelengths (normal photoperiod, continuous illumination, continuous darkness, and yellow and green light during the night). The objective of this study was to determine whether variation in light conditions could affect the behavior of tea green leafhopper adult females and males.

## Materials and Methods

### Insect rearing

The leafhoppers were collected from a tea plantation (Fuzhou, Fujian Province, China, 119.2°E, 26.1°N) belonging to the Fujian Agriculture and Forestry University (FAFU) (We received permission from FAFU to conduct this study). We cut several tea branches with leaves that contained eggs of *E*. *onukii*. The branches were brought back to the laboratory and the stems were immersed in water under conditions of 12:12 photoperiod (7:00–19:00 light by quartz lamp, 19:00–7:00 with darkness, China standard time), 27±1°C. The light intensity during the day was around 2000 lux (see S1 Fig for spectral characteristics of the quartz lamp). Once the eggs hatched, nymphs were monitored until they reached developmental stages (instars) 4–5. They were then carefully transferred into a tube containing fresh tea tips until their emergence as adults. The new adults were sexed and then used in the following experiments.

### Video recording system

**Fig 1:**
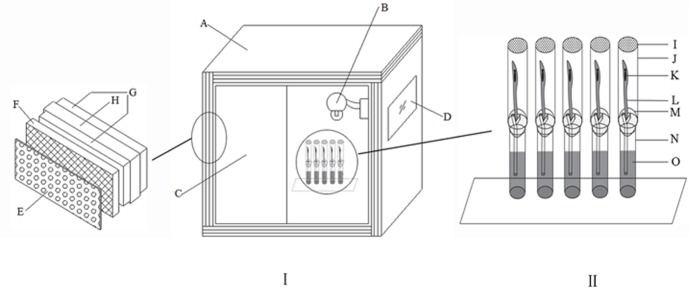
System used for leafhopper behavior observations. A: observation room; B: infrared camera (two in total, the figure shows one); C: door; D: observation window (hollow glass); E: deadening felt (inside the room); F: copper mesh; G: composite board; H: sound proof cotton; I: plastic wrap (with ventholes); J: glass tube B (10mm in diameter, 100mm in high); K,S: tested leafhopper; L: tea tip; M: cotton; N: glass tube B.

The *E*. *onukii* adult has a mean body length (with wings) of approximately 2.5 mm, which makes behavioral activities difficult to be observed. A new system was designed to observe the behavior of the leafhoppers under the various light treatments, in which tubes containing newly emerged adults were placed in the center of an observation chamber (1.2 m × 1.2 m × 1.2 m, Fig 1). The observation chamber contained two high-speed infrared cameras (1/4 SONY CCD, 24 zoom, fast automatic focusing, with cradle control) were positioned on the two opposite sides of the chamber. To reduce outside noise and vibrations, the chamber walls were soundproofed with cotton pressed between two wooden planks (each 5cm thick). To further reduce noise and the interferences of radio waves, the interior of the room was covered with a black sound deadening felt maintained in place with copper wire mesh (100 meshes per inch) (see Fig 1 for details). The temperature of the observation chamber was kept constant at 27±1°C.

### Behavioral observations

Five different light treatments were used: A) same photoperiod as the rearing conditions, i.e. 12 hours of normal illumination (called Period 1 (day) between 7:00–19:00) and 12 hours of darkness (Period 2 (night) between 19:00–7:00), representing the normal conditions in the field (LD); B) 24 h of continuous illumination (LL); C) 24 h of continuous darkness (DD); D) Period 1 with normal illumination (quartz lamp) and Period 2 illuminated with yellow LED light (dominant wavelength: 569.0 nm, see S2 Fig for details) (LY); and E) Period 1 with normal illumination (quartz lamp) and Period 2 illuminated with green LED light (dominant wavelength: 508.0 nm, see S3 Fig for details) (LG). Light intensity remained constant at 2000 lux.

Each treatment included 20 replicates, i.e. 10 virgin adult females and 10 virgin adult males, which had emerged within 12 hours. For each observation session, only one newly emerged (within 12 h) adult was transferred into a tube (diameter 1cm, height 10cm, made of transparent borosilicate glass, which displays high transmission of light) containing a tea tip maintained alive with water at the bottom (see Fig 1). The tea tips were picked from the tea plants in the greenhouse, not previously exposed to tea green leafhopper. The tea tips were replaced every other day. Very few leafhoppers (2 females and 5 males) were found dead during the experiments; in those cases, data were discarded and new leafhoppers were used to obtain 10 replicates per treatment.

The cameras began continuous recording at 0:00 h of the second (one day acclimation) day for the next 10 consecutive days. To analyze the frequencies and lengths of the different types of behavior, the videos were repetitively played using various speeds (maximum eightfold). The three types of behavior were reported as follows: 1) the number of locomotion events per hour, including walking, jumping, and flying. A locomotion event was considered as a fast move with the distance being equal or longer to the body length of the tested leafhopper, 2) the number of cleaning events per hour, where leafhoppers used their legs to clean their body, mainly their head and wings, 3) the number of minutes of searching activities per hour, which was described as any time a leafhopper left the tea tip and stayed or moved along the tube, as if it was to searching something (such as mate, new food, etc.).

### Data analyses

For each treatment and sex, charts of activities per hour were produced, and then the total number of activities (locomotion and cleaning) or minutes of searching activities over each Period (1 (day) and 2(night)) and for 24 hours was calculated for each of the 10 days. Data were compared among different photoperiod treatments (Treatment A, B and C) and wavelength treatments (Treatment B, D and E). Repeated-measures analyses of variance were used to determine whether the variation over the 10 days was significant (within subject variation) and if these results significantly differed among treatments and sexes (between subject variation). To examine the potential effect of light on day versus night activities, a ratio for each type of activities was calculated (Period 1 activities / total activities for a period of 24 hours) followed by repeated-measures analysis of variance. Normality and homogeneity of variances were satisfied and since the chamber effect as experimental run was *a priori* tested and was not significant, it was not further carried as a variable in the analyses.

Finally, the total number of activities (locomotion and cleaning) or minutes of searching activities in Periods 1 and 2 over the ten days was calculated for each individual. Two-way analysis of variance was used to compare treatment (photoperiod and wavelength separately), sex and their interaction.

## Results

Overall, the different light conditions resulted in significant changes in behavioral activities during the day and night as well as circadian patterns. Line charts of the mean values of locomotion, cleaning, and searching activities for each hour of the day for the 10 days of experimentation are shown in S4, S5 and S6 Figs. In the following sections, we described the general trends of behavioral patterns under the various photoperiods (LD, LL and DD) and wavelength treatments (LL, LY and LG).

### The effects of photoperiod

#### Locomotion behavior

**Fig 2:**
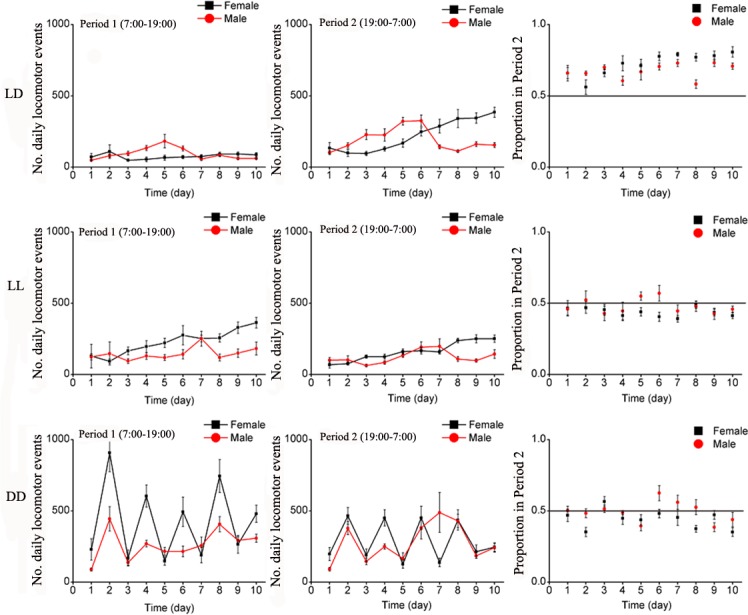
Mean number of daily locomotion events of *E*. *onukii* adults under varying photoperiod treatments for the 10 days of observation, during Period 1 (left side) and Period 2 (center graphs), and the average proportion of locomotion events in Period 2 (Period 1 / (Period 1 + Period 2), right side). Each day (24H) was divided as Period 1(7:00–19:00) and Period 2(19:00–7:00). LD: Period 1 with light of quartz lamp, Period 2 with darkness; LL: continuous illumination by quartz lamp; DD: continuous darkness.

Under LD, LL and DD, locomotion activities and the proportion of locomotion activities in Period 2 (Period 2 / (Period 1 + Period 2)) of both *E*. *onukii* males and females significantly varied over the 10 days and among sexes and treatments (Table 1). Under normal light condition (LD), leafhoppers moved little during the day (Period 1) (Fig 2). At night, the insects were more active and values tended to increase over the 10 day-period, especially for males on Day 5.

**Table 1:** Comparison of the behaviors of *E*. *onukii*, a) locomotion, b) cleaning, and c) searching, over time, sex and photoperiod treatments (LD, LL, DD) using a two factor repeated measures analysis of variance.

Source of variation	Daily amount			Daily ratio of Period 2		
	df	*F*-value	*P*-value	df	*F*-value	*P*-value
**a) Locomotion events**						
**Within subject variation**						
Day	9, 46	15.59	<0.001	9, 46	2.36	0.013
Day*Treatment	18, 94	12.48	<0.001	18, 94	3.32	<0.001
Day*Sex	9, 46	5.16	<0.001	9, 46	2.02	0.037
Day*Treatment*Sex	18, 94	5.33	<0.001	18, 94	2.06	0.007
**Between subject variation**						
Treatment	2, 54	43.54	<0.001	2, 54	259.91	<0.001
Sex	1, 54	10.38	0.002	1, 54	2.08	0.155
Treatment*Sex	2, 54	2.18	0.123	2, 54	10.68	<0.001
**b) Cleaning events**						
**Within subject variation**						
Day	9, 46	4.99	0.001	9, 46	0.87	0.541
Day*Treatment	18, 94	7.26	<0.001	18, 94	0.77	0.721
Day*Sex	9, 46	2.55	0.039	9, 46	2.24	0.023
Day*Treatment*Sex	18, 94	4.75	<0.001	18, 94	1.25	0.225
**Between subject variation**						
Treatment	2, 54	66.63	<0.001	2, 54	77.59	<0.001
Sex	1, 54	0.41	0.527	1, 54	0.01	0.924
Treatment*Sex	2, 54	11.34	<0.001	2, 54	0.83	0.443
**c) Searching duration**						
**Within subject variation**						
Day	9, 46	18.53	<0.001	9, 46	3.74	<0.001
Day*Treatment	18, 94	6.13	<0.001	18, 94	3.31	0.003
Day*Sex	9, 46	2.52	0.015	9, 46	2.06	0.039
Day*Treatment*Sex	18, 94	5.89	<0.001	18, 94	3.12	<0.001
**Between subject variation**						
Treatment	2, 54	13.60	<0.001	2, 54	45.23	<0.001
Sex	1, 54	40.78	<0.001	1, 54	0.31	0.578
Treatment*Sex	2, 54	1.90	0.159	2, 54	2.57	0.086

Treatments: A) Period 1 with light of quartz lamp, Period 2 with darkness; B) continuous illumination by quartz lamp; C) continuous darkness. Each day (24H) was divided as Period 1(7:00–19:00) and Period 2(19:00–7:00). Repeated-measures ANOVAs were used, since the sphericity assumption was not satisfied (*P*<0.05), Huynh-Feldt was used for adjusting.

The locomotion behavior of leafhopper was greatly affected by changes in light condition. Under continuous illumination (LL), both males and females were continuously active and activities slightly increased over time (Fig 2). However under complete darkness (DD), the rhythm of locomotion activities completely changed with a two-day cycle pattern and females showed a stronger signal than males (Fig 2).

**Fig 3:**
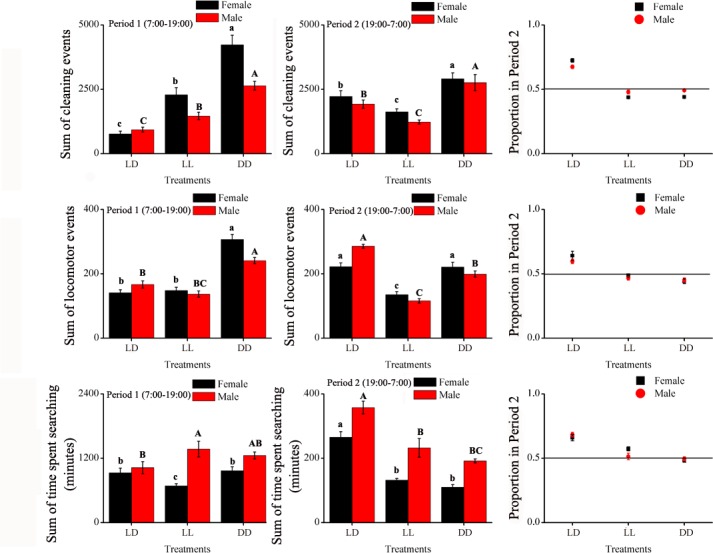
Total amount of the three behaviors, locomotion, cleaning and searching activities, for the 10 days during Period 1 (left side) or Period 2 (center) of *E*. *onukii* males and females total ratio in Period 2 (Period 1 / (Period 1 + Period 2), right side), under varying photoperiod treatments. Significant differences among the three treatments are marked with different letters (LSD, *P*<0.05, females marked with lowercase letters, males marked with capital letters).

The total numbers of locomotion events over 10 days were calculated for each sex and treatment. In Period 1, although there were significant differences between males and females, both were the most active in complete darkness (DD), followed by continuous illumination and finally normal photoperiod (Table 2, Fig 3). In Period 2, only treatment effect was significant (Table 2) with more locomotion events in DD than LD and LL. Overall, both males and females were more active during the night than during the day under normal photoperiod compared to LL and DD (Fig 3).

**Table 2:** Results of a two-way ANOVA comparing the total number of locomotion and cleaning events and total amount of searching activities over the 10 days between sexes and treatments for a) Period 1and b) Period 2.

Period	Source of variation	Locomotion events			Cleaning events			Searching duration		
		df	*F*-value	*P*-value	df	*F*-value	*P*-value	df	*F*-value	*P*-value
Period 1	Treatment	2, 54	70.60	<0.001	2, 54	86.76	<0.001	2, 54	1.03	0.363
	Sex	1, 54	17.51	<0.001	1, 54	3.58	0.064	1, 54	21.598	<0.001
	Treatment*Sex	2, 54	8.11	<0.001	2, 54	8.67	0.001	2, 54	5.19	0.009
Period 2	Treatment	2, 54	24.50	<0.001	2, 54	81.62	<0.001	2, 54	52.29	<0.001
	Sex	1, 54	2.99	0.089	1, 54	0.74	0.393	1, 54	45.15	<0.001
	Treatment*Sex	2, 54	0.18	0.831	2, 54	11.31	<0.001	2, 54	0.16	0.853

Treatments: A) Period 1 with light of quartz lamp, Period 2 with darkness; B) continuous illumination by quartz lamp; C) continuous darkness. Each day (24H) was divided as Period 1 (7:00–19:00) and Period 2 (19:00–7:00). Two-way ANOVAs were used.

#### Cleaning Behavior

**Fig 4:**
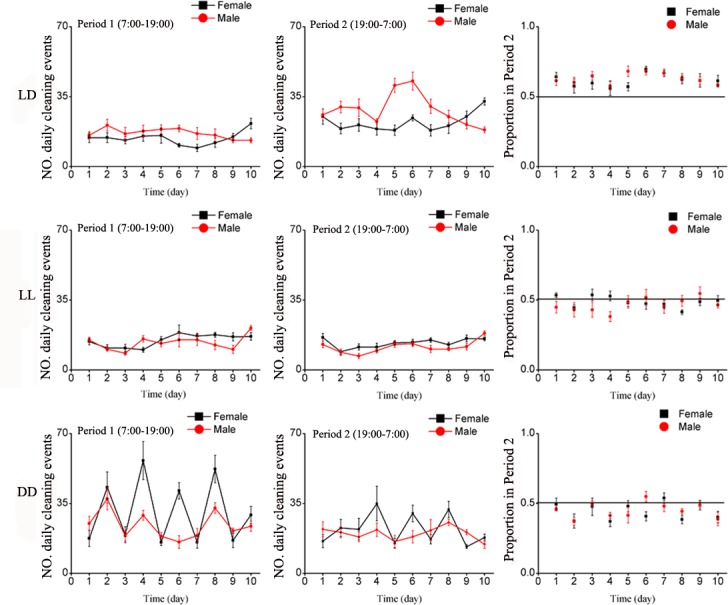
Mean number of daily cleaning events of *E*. *onukii* adults under varying photoperiod treatments for the 10 days of observation, during Period 1 (left side) and Period 2 (center graphs), and the average proportion of locomotion events in Period 2 (Period 1 / (Period 1 + Period 2), right side). Each day (24H) was divided as Period 1(7:00–19:00) and Period 2(19:00–7:00). LD: Period 1 with light of quartz lamp, Period 2 with darkness; LL: continuous illumination by quartz lamp; DD: continuous darkness.

Both males and females exhibited similar cleaning patterns over the period of 10 days and among treatments, (Table 1). In normal light condition (LD), the number of cleaning events was greater during Period 2 than Period 1 (Fig 4).

Under continuous illumination (LL), both males and females exhibited constant cleaning behavior for both Periods 1 and 2 (Fig 4). In continuous darkness (DD), females and males (however to a lesser extent) exhibited a pattern of activities with peaks occurring every 48 hours. Females, however, tended to clean themselves more in Period 1 than in Period 2 (Fig 4). Overall, the total number of cleaning events over the 10-day period was significantly affected by treatment. The effects of various treatments however, were inconsistent between sexes as reflected in a significant treatment-by-sex interaction, while sex was not significant (Table 2). In Period 1, leafhoppers were more active cleaning in DD than LD and LL. Like locomotion, under normal photoperiod (LD), both males and females were cleaning more in Period 2 than when exposed to LL or DD (Fig 3).

#### Searching behavior

**Fig 5:**
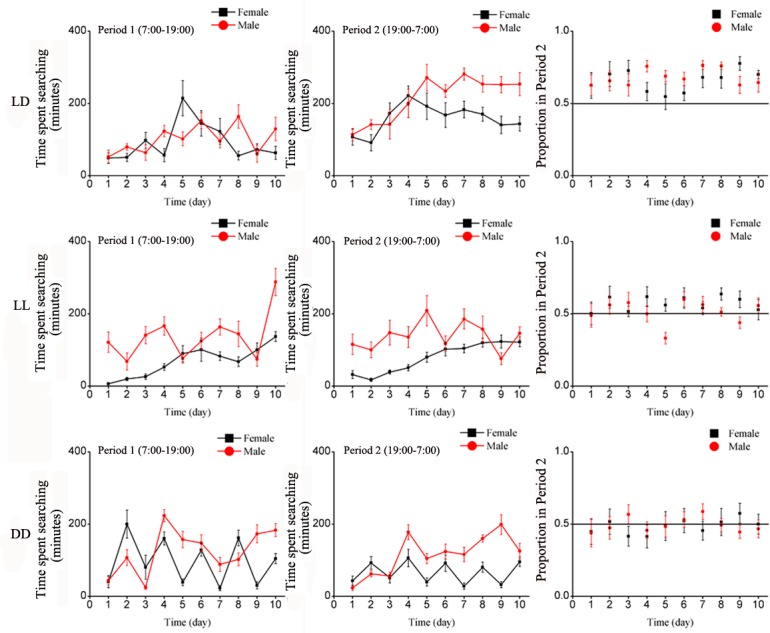
Mean number of daily searching events of *E*. *onukii* adults under varying photoperiod treatments for the 10 days of observation, during Period 1 (left side) and Period 2 (center graphs), and the average proportion of locomotion events in Period 2 (Period 1 / (Period 1 + Period 2), right side). Each day (24H) was divided as Period 1(7:00–19:00) and Period 2(19:00–7:00). LD: Period 1 with light of quartz lamp, Period 2 with darkness; LL: continuous illumination by quartz lamp; DD: continuous darkness.

Searching behavior of both males and females was significantly influenced by the variation in the type of wavelength illumination during Period 2 i.e. at night (Table 1). Under LD, the searching behavior of both males and females greatly varied over time. The time spent searching at night (Period 2) tended to be greater than in Period 1, especially for males (Fig 5). Under LL, females were less active than males although the searching activities tended to progressively increased over time. Females exposed to DD exhibited a two-day pattern of activities with higher peaks in Period 1 than Period 2. Overall, like for locomotion and cleaning, under normal photoperiod, both males and females tended to be more active searching during Period 2 than Period 1 (Fig 3). Overall, the total number of searching behavior over the 10-day period was not significantly affected by treatment, however, there was a significant treatment-by-sex interaction (Table 2).

### The effects of light wavelength

#### Locomotion behavior

**Fig 6:**
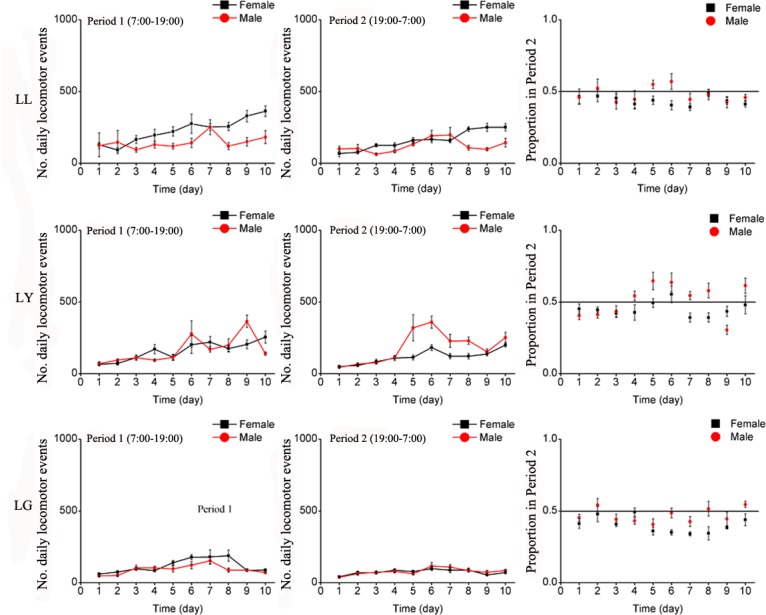
Mean number of daily locomotion events of *E*. *onukii* adults under varying wavelength treatments for the 10 days of observation, during Period 1 (left side) and Period 2 (center graphs), and the average proportion of locomotion events in Period 2 (Period 1 / (Period 1 + Period 2), right side). Each day (24H) was divided as Period 1(7:00–19:00) and Period 2(19:00–7:00). LL: continuous illumination by quartz lamp; C) continuous darkness; LY: Period 1 with light of quartz lamp, Period 2 with yellow light (LED); LG: Period 1 with light of quartz lamp, Period 2 with green light (LED).

Changes in wavelength at night (Period 2) significantly affected the locomotion behavior of *E*. *onukii* (Table 3). Compared to LL, locomotion of leafhoppers exposed to LY and LG was suppressed and this was especially true in LG (Fig 6). Under the three treatments, the number of locomotion events during Period 1 was relatively similar to those in Period 2 (Fig 6).

**Table 3:** Comparison of the behaviors of *E*. *onukii*, a) locomotion, b) cleaning, and c) searching, over time, sex and wavelength treatments (LL, LY, LG) using a two way repeated measures analysis of variance.

Source of variation	Daily amount			Daily ratio of Period 2		
	df	*F*-value	*P*-value	df	*F*-value	*P*-value
**a) Locomotion events**						
**Within subject variation**						
Day	9, 46	26.97	<0.001	9, 46	4.03	<0.001
Day*Treatment	18, 94	4.11	<0.001	18, 94	3.20	<0.001
Day*Sex	9, 46	2.39	0.033	9, 46	3.19	0.001
Day*Treatment*Sex	18, 94	3.91	<0.001	18, 94	1.57	0.067
**Between subject variation**						
Treatment	2, 54	17.66	<0.001	2, 54	5.14	0.009
Sex	1, 54	1.50	0.288	1, 54	24.28	<0.001
Treatment*Sex	2, 54	6.15	0.004	2, 54	0.51	0.603
**b) Cleaning events**						
**Within subject variation**						
Day	9, 46	14.28	<0.001	9, 46	1.68	0.105
Day*Treatment	18, 94	3.42	<0.001	18, 94	1.35	0.168
Day*Sex	9, 46	2.45	0.028	9, 46	1.70	0.102
Day*Treatment*Sex	18, 94	3.36	<0.001	18, 94	0.99	0.470
**Between subject variation**						
Treatment	2, 54	8.78	<0.001	2, 54	12.61	<0.001
Sex	1, 54	0.57	0.453	1, 54	0.25	0.622
Treatment*Sex	2, 54	4.14	0.021	2, 54	0.64	0.533
**c) Searching duration**						
**Within subject variation**						
Day	9, 46	37.00	<0.001	9, 46	1.47	0.177
Day*Treatment	18, 94	3.64	<0.001	18, 94	3.66	<0.001
Day*Sex	9, 46	2.47	0.026	9, 46	1.36	0.219
Day*Treatment*Sex	18, 94	2.76	0.002	18, 94	3.04	<0.001
**Between subject variation**						
Treatment	2, 54	4.42	0.017	2, 54	2.57	0.086
Sex	1, 54	8.29	0.006	1, 54	1.21	0.276
Treatment*Sex	2, 54	12.02	<0.001	2, 54	2.00	0.146

Treatments: B) continuous illumination by quartz lamp; D) Period 1 with light of quartz lamp, Period 2 with yellow light (LED); E) Period 1 with light of quartz lamp, Period 2 with green light (LED). Each day (24H) was divided as Period 1(7:00–19:00) and Period 2(19:00–7:00). Repeated-measures ANOVAs were used, since the sphericity assumption was not satisfied (*P*<0.05), Huynh-Feldt was used for adjusting.

**Fig 7:**
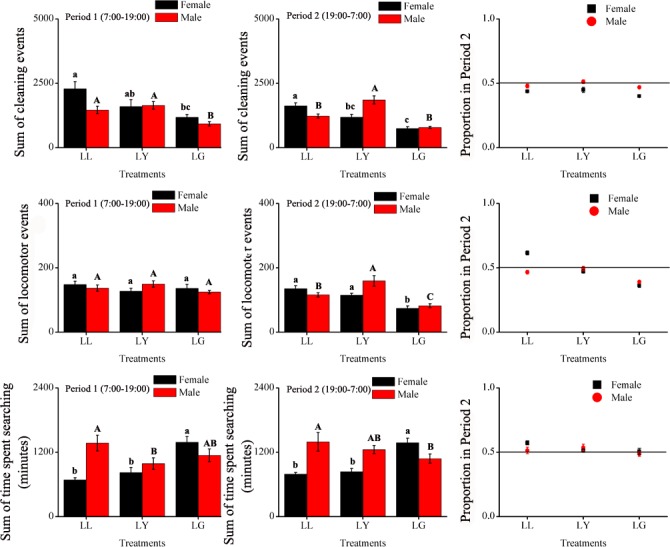
Total amount of the three behaviors, locomotion, cleaning and searching activities, for the 10 days during Period 1 (left side) or Period 2 (center) of *E*. *onukii* males and females total ratio in Period 2 (Period 1 / (Period 1 + Period 2), right side), under varying wavelength treatments. Significant differences among the three treatments are marked with different letters (LSD, *P*<0.05, females marked with lowercase letters, males marked with capital letters).

Locomotion behavior of both males and females was statistically non-significant when summed over the 10 days showing similar suppression of activities in Period 2 (Table 4). The overall number of locomotion events under LG was less lower than those under LL and LY (Fig 7).

**Table 4:** Comparison of the three behavioral values of *E*. *onukii* in total ten days’ observation in different light wavelength treatments, during Period 1and Period 2 using a two way ANOVA.

Period	Source of variation	Locomotion events			Cleaning events			Searching duration		
		df	*F*-value	*P*-value	df	*F*-value	*P*-value	df	*F*-value	*P*-value
Period 1	Treatment	2, 54	10.29	<0.001	2, 54	0.83	0.443	2, 54	1.03	0.363
	Sex	1, 54	5.16	0.027	1, 54	0.001	0.977	1, 54	21.60	<0.001
	Treatment*Sex	2, 54	2.80	0.070	2, 54	2.01	0.144	2, 54	5.19	0.009
Period 2	Treatment	2, 54	32.52	<0.001	2, 54	21.98	<0.001	2, 54	5.87	0.005
	Sex	1, 54	1.56	0.217	1, 54	2.07	0.156	1, 54	5.41	0.024
	Treatment*Sex	2, 54	13.70	<0.001	2, 54	5.84	0.005	2, 54	9.51	<0.001

Treatments: B) continuous illumination by quartz lamp; D) Period 1 with light of quartz lamp, Period 2 with yellow light (LED); E) Period 1 with light of quartz lamp, Period 2 with green light (LED). Each day (24H) was divided as Period 1 (7:00–19:00) and Period 2 (19:00–7:00). Two-way ANOVAs were used.

#### Cleaning behavior

**Fig 8:**
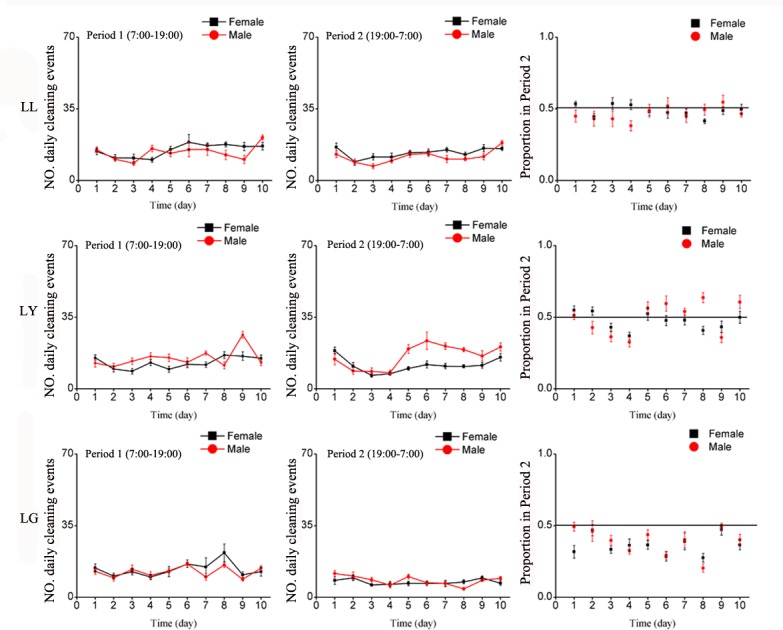
Mean number of daily cleaning events of *E*. *onukii* adults under varying wavelength treatments for the 10 days of observation, during Period 1 (left side) and Period 2 (center graphs), and the average proportion of locomotion events in Period 2 (Period 1 / (Period 1 + Period 2), right side). Each day (24H) was divided as Period 1(7:00–19:00) and Period 2(19:00–7:00). LL: continuous illumination by quartz lamp; C) continuous darkness; LY: Period 1 with light of quartz lamp, Period 2 with yellow light (LED); LG: Period 1 with light of quartz lamp, Period 2 with green light (LED).

The number of cleaning events significantly varied over the period of 10 days and among treatments, especially for Period 2 (LY) (Table 3, Fig 8). Cleaning activities under LG were suppressed in both Periods compared to LL and LY (Fig 7). Under LG leafhoppers exhibited less cleaning activities during Period 2 than under LL and LY (Fig 8). The total number of activities calculated over the ten days showed significant treatment and sexes by treatment interaction effects in Period 2 (Table 4), which was reflected being less active during Period 2 than Period 1, especially for the males.

#### Searching behavior

**Fig 9:**
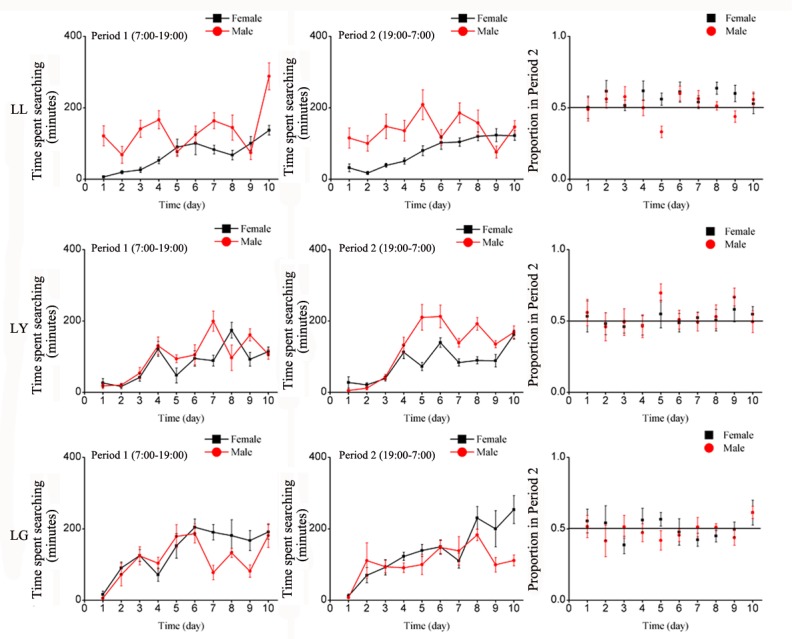
Mean number of daily searching events of *E*. *onukii* adults under varying wavelength treatments for the 10 days of observation, during Period 1 (left side) and Period 2 (center graphs), and the average proportion of locomotion events in Period 2 (Period 1 / (Period 1 + Period 2), right side). Each day (24H) was divided as Period 1(7:00–19:00) and Period 2(19:00–7:00). LL: continuous illumination by quartz lamp; C) continuous darkness; LY: Period 1 with light of quartz lamp, Period 2 with yellow light (LED); LG: Period 1 with light of quartz lamp, Period 2 with green light (LED).

Unlike in LL, under LY and LG, females and males took a few days to become more active in searching but then the behavior pattern fluctuated in a similar way than under LL (Table 3). Searching behavior greatly varied during the course of the 10 days but with no or little significant differences among treatments and sexes (Fig 9 and Table 4).

## Discussion

Our study showed that both photoperiod and light wavelength had significant effects on locomotion, cleaning and searching behaviors of *E*. *onukii* adults. A few studies on other leafhopper species have reported changes in behavior under changing light conditions. In *Graminella nigrifrons*, the males oriented toward light when searching for mates [26]. *Dalbulus maidis* displays more phototaxis to 560 nm than any other wavelength [27].

The first important observation was that *E*. *onukii* displayed more active at night than during the day and could be considered either day neutral or slightly nocturnal. *E*. *onukii* reproduction is a long process that mainly occurs at night, possibly to reduce predation (Shi et al. in preparation). Unlike some other leafhoppers such as *Macrosteles fascifrons* and *G*. *nigrifrons* [28, 29], *E*. *onukii* does not migrate in daytime and can feed on the evergreen tea plants during the whole year (although populations and activities may be reduced during cooler winter conditions, suggesting possible diapause), it is possible that nocturnal activities represent an adaptive response to avoid predators, especially during reproductive periods.

Circadian rhythms have been detected in most organisms including insects [30, 31]. They can influence physiology and behavior such as locomotion (walking, flying, swimming, etc.), feeding, cleaning, courtship, and mating [18, 32, 33], and are regulated by clock genes that maintain cycles of around 24 hours. They can be influenced by external stimuli that reset the rhythm to a new period [32, 34]. Among these stimuli, light is considered one of the most important factors for insects [3, 35]. Continuous illumination can lead to behavioral arrhythmicity in insect such as *D*. *melanogaster* and *Calliphora vicina* [36, 37]. In our case, under complete darkness, the species modified its rhythm to a two-day cycle of activities.

Insects have different types of photoreceptors that are distributed in various tissues, leading to different levels of sensitivity to wavelengths [3, 38, 39]. Changes in wavelength trigger responses of the photoreceptors, which may affect physiology and behaviors. In our case, different wavelength exposure in Period 2 led to significant behavioral changes. For instance, under LG, locomotion and cleaning activities were seriously suppressed when compared to LL and LY.

Our study also showed that changes in behavior in function of photoperiod and wavelength could be sex-specific. Between-sex difference has already been observed in *E*. *onukii* where male adults are more attracted by yellow sticky cards than females [16]. We also observed that males were more active than females only under the yellow light treatment suggesting phototaxis response to this wavelength. Sexual dimorphism in phototaxis has been shown in other leafhoppers, such as *E*. *vitis* and *Scaphoideus titanus* [40, 41]. Differences found between sexes may be attributed to sexual dimorphism of their photoreceptors leading to variation in response to different wavelengths [42]. Insects such as butterflies (e.g. species of *Lycaena* and *Colias erate*) and *D*. *melanogaster* display such sexual dimorphism in response to wavelength variation [43, 44, 45]. Further research is needed to confirm the differences in photoreceptors between male and female leafhoppers.

We have to acknowledge that this experiment was conducted under controlled laboratory conditions with rectangular light cycles and under constant temperature. This does not necessarily represent what leafhoppers experience in their natural environment where photoperiod and temperature can vary over the day and year. Experiments conducted with *D*. *melanogaster* under natural or semi-natural conditions show that additional daytime peaks can be found and be of importance [46, 47]. Moreover, some strains of *D*. *melanogaster* have been reported not to be day-neutral as first thought but exhibit an ovarian diapause under long nights and low temperatures [18]. *E*. *onukii* populations in China may also be influenced by these conditions. Previous observations show that populations tend to peak in July, when temperatures are 26°C or higher, and decline in the fall when temperatures drop to 15°C [16, 48]. Future studies should examine how natural photoperiod and temperature affect *E*. *onukii* behavioral patterns. Since photoperiodic responses can be complex as they involve several factors such as photoreception mechanisms, night length influence, and endocrine effectors that determine developmental steps, further genetic and molecular analyses should be included to better understand the roles of clock genes in the photoperiodic responses of the species [18].

Several of the 22,000 species of leafhoppers in the world are considered serious pests in agriculture and forestry [49]. Besides insecticides (including microbial insecticides),yellow sticky cards, natural enemies, and trap plants have been used as alternative management approaches [50, 51]. A previous study suggests that *E*. *onukii* male adults tend to be more attracted to yellow sticky cards than females [16]. Bӧll et al. (2004) also suggest that the difference in activity patterns between the two sexes of *E*. *vitis* may influence the level of attractiveness to the color of sticky card traps [40]. Considering that the present study showed that most behavioral activities such as locomotion and searching occur at night, to enhance pest control efficiency, it may be useful to supplement the control of sticky cards that are used during the day with a light control system at night. Although, light traps are now widely used for pest control [6, 52, 53], negative impacts on natural pest enemies remain a concern [54, 55]. Further research will be required to test such control systems to determine whether natural pest enemies, such as spiders, parasitoids, predatory mites, are also trapped leading to a decline in their populations as well as the pest. Further experiments of these lights (with or without trap) on leafhopper populations and pest enemies in the field will help determine their effectiveness as alternative control.

## Supporting Information

S1 Fig Spectral distribution of quartz lamp. (TIF)

S2 Fig Spectral distribution of yellow LED light. (TIF)

S3 Fig Spectral distribution of green LED light. (TIF)

S4 Fig Line charts of the mean values of locomotion events (Mean ± SEM). Locomotion events (with standard errors) at each hour of the day for the 10 days of experimentation. For each hour, the mean values of locomotion behaviour of 10 tested leafhoppers in each treatment are shown by line charts with standard errors. Every small chart got the same axis values. Treatments: A) Period 1 with light of quartz lamp, Period 2 with darkness; B) continuous illumination by quartz lamp; C) continuous darkness; D) Period 1 with light of quartz lamp, Period 2 with yellow light (LED); E) Period 1 with light of quartz lamp, Period 2 with green light (LED). Each day (24H) was divided as Period 1(7:00–19:00) and Period 2(19:00–7:00).
(TIF)

S5 Fig Line charts of the mean values of cleaning events (Mean ± SEM). Cleaning events (with standard errors) at each hour of the day for the 10 days of experimentation. For each hour, the mean values of cleaning behaviour of 10 tested leafhoppers in each treatment are shown by line charts with standard errors. Every small chart got the same axis values. Treatments: A) Period 1 with light of quartz lamp, Period 2 with darkness; B) continuous illumination by quartz lamp; C) continuous darkness; D) Period 1 with light of quartz lamp, Period 2 with yellow light (LED); E) Period 1 with light of quartz lamp, Period 2 with green light (LED). Each day (24H) was divided as Period 1(7:00–19:00) and Period 2(19:00–7:00).
(TIF)

S6 Fig Line charts of the mean values of searching duration (Mean ± SEM). Searching duration (with standard errors) at each hour of the day for the 10 days of experimentation. For each hour, the mean values of searching behaviour of 10 tested leafhoppers in each treatment are shown by line charts with standard errors. Every small chart got the same axis values. Treatments: A) Period 1 with light of quartz lamp, Period 2 with darkness; B) continuous illumination by quartz lamp; C) continuous darkness; D) Period 1 with light of quartz lamp, Period 2 with yellow light (LED); E) Period 1 with light of quartz lamp, Period 2 with green light (LED). Each day (24H) was divided as Period 1(7:00–19:00) and Period 2(19:00–7:00).
(TIF)

## References

[1] Beck SD (Ed) (1980) Insect photoperiodism, 2^nd^ ed New York: Academic Press.

[2] Endler JA (1987) Predation, light intensity and courtship behavior in *Poecilia reticulata* (Pisces: Poeciliidae). Anim Behav 35: 1376–1385.

[3] Saunders D (2012) Insect photoperiodism: seeing the light. Physiol Entomol 37: 207–218.

[4] Adkisson PL (1964) Action of the photoperiod in controlling insect diapause. Amer Naturalist 98: 357–374.

[5] Geffen KG, Grunsven RH, Ruijven J, Berendse F, Veenendaal EM (2014) Artificial light at night causes diapause inhibition and sex-specific life history changes in a moth. Ecol Evol 4: 2082–2089. 10.1002/ece3.1090 25360250 PMC4201423

[6] Shimoda M, Honda KI (2013) Insect reactions to light and its applications to pest management. Appl Entomol Zool 48: 413–421.

[7] Wang S, Tan XL, Michaud JP, Zhang F, Guo X (2013) Light intensity and wavelength influence development, reproduction and locomotion activity in the predatory flower bug *Orius sauteri* (Poppius) (Hemiptera: Anthocoridae). BioControl 58: 667–674.

[8] Whittaker MS, Kirk WDJ (2004) The effect of photoperiod on walking, feeding, and oviposition in the western flower thrips. Entomol Exp Appl 111:209–214.

[9] Grossfield J (1971) Geographic distribution and light-dependent behavior in *Drosophila*. Proc Nat Acad Sci USA 68: 2669–2673. 5288242 10.1073/pnas.68.11.2669 PMC389497

[10] Diaz-Fleischer F, Arredondo J (2011) Effects of post-eclosion light-dark regimes on mating performance of mass-reared tephritid fly *Anastrepha ludens*. Entomol Exp Appl 141: 52–58.

[11] Coombe PE (1981) Wavelength specific behavior of the whitefly *Trialewodes vaporariorum* (Homoptera: Aleyrodidae). J Comp Physiol 144: 83–90.

[12] Omkar G, Mishra G, Singh K (2005) Effects of different wavelengths of light on the life attributes of two aphidophagous ladybirds (Coleoptera: Coccinellidae). Eur J Entomol 102: 33–37.

[13] Wang S, Wang K, Michaud J, Zhang F, Tan XL (2014) Reproductive performance of *Propylea japonica* (Coleoptera: Coccinellidae) under various light intensities, wavelengths and photoperiods. Eur J Entomol 111: 341–347.

[14] Qin D, Zhang L, Xiao Q, Dietrich C, Matsumura M (2015) Clarification of the identity of the tea green leafhopper based on morphological comparison between Chinese and Japanese specimens. PloS ONE 10: e0139202 10.1371/journal.pone.0139202 26422616 PMC4589377

[15] Shi LQ, Lin MZ, Chen LL, Lin MQ, Vasseur L, You MS (2014) Changing the specific name of tea green leafhoppers in major tea plantations in Fujian Province, China. Journal of Fujian Agriculture and Forestry University (Natural Science Edition) 43: 456–459.

[16] Shi LQ, Zeng ZH, Huang HS, Zhou YM, Vasseur L, You MS (2015) Identification of *Empoasca onukii* (Hemiptera: Cicadellidae) and monitoring of its populations in the tea plantations of south China. J Econ Entomol 108: 1025–1033. 10.1093/jee/tov054 26470225

[17] Muraleedharan N, Chen Z (1997) Pests and diseases of tea and their management. J Plant Crops 25: 15–43.

[18] Zhang W, Lin M, Zhang H (1997) Relationship between temperature and development of *Emapoasca vitis* Gothe (Lepidoptera: Cicadelidae). J. Anhui. Agric. Univ. 24: 332–335.

[19] Saunders DS, Bertossa R (2011) Deciphering time measurement: the role of circadian ‘clock’ genes and formal experimentation in insect photoperiodism. J Insect Physiol 57: 557–566. 10.1016/j.jinsphys.2011.01.013 21295039

[20] Rakitov RA (2000) Secretion of brochosomes during the ontogenesis of a leafhopper, *Oncometopia orbona* (F.) (Insecta, Homoperta, Cicadellidae). Tissue Cell 32: 29–39. 10.1054/tice.1999.0084 10798315

[21] Rakitov RA (2004) Powdering of egg nests with brochosomes and related sexual dimorphism in leafhoppers (Hemiptera: Cicadellidae). Zool J Linn Soc 140: 353–381.

[22] Reilly SM, Mcelroy EJ, Biknevicius AR (2007) Posture, gait and the ecological relevance of locomotion costs and energy-saving mechanisms in tetrapods. Zool 110: 271–289. 10.1016/j.zool.2007.01.003 17482802

[23] Bonsignori G, Stefanini C, Scarfogliero U, Mintchev S, Benelli G, Dario P (2013) The green leafhopper, *Cicadella viridis* (Hemiptera, Auchenorrhyncha, Cicadellidae), jumps with near-constant acceleration. J Exp Biol 216: 1270–1279. 10.1242/jeb.076083 23487271

[24] Saxena K, Kumar H (1980) Interruption of acoustic communication and mating in a leafhopper and a planthopper by aerial sound vibrations picked up by plants. Experientia 36: 933–936.

[25] Hunt RE, Nault LR (1991) Roles of interplant movement, acoustic communication, and phototaxis in mate-location behavior of the leafhopper *Graminella nigrifrons*. Behav Ecol Sociobiol 28: 315–320.

[26] Hunt RE, Morton TL (2001) Regulation of chorusing in the vibrational communication system of the leafhopper *Graminella nigrifrons*. American Zoologist 41: 1222–1228.

[27] Todd J, Phelan P, Nault L (1990) Orientation of the leafhopper, *Dalbulus maidis* (Homoptera: Cicadellidae), to different wavelengths of reflected light. J Insect Behav 3: 567–571.

[28] Lopes J, Nault L, Phelan P (1995) Periodicity of diel activity of *Graminella nigrifrons* (Homoptera: Cicadellidae) and implications for leafhopper dispersal. Ann Entomol Soc Amer 88: 227–233.

[29] Wallis R (1962) Spring migration of the six-spotted leafhopper in the western Great Plains. J Econ Entomol 55: 871–874.

[30] Harker J (1973) Circadian rhythms in insects In: Mills JN, editor. Biological aspects of circadian rhythms: Springer US pp. 189–233

[31] Bloch G, Hazan E, Rafaeli A (2013) Circadian rhythms and endocrine functions in adult insects. J Insect Physiol 59: 56–69. 10.1016/j.jinsphys.2012.10.012 23103982

[32] Saunders DS (1997) Insect circadian rhythms and photoperiodism. Invertebrate Neuroscience 3: 155–164. 9783440 10.1007/BF02480370

[33] Tomioka K, Uryu O, Kamae Y, Umezaki Y, Yoshii T (2012) Peripheral circadian rhythms and their regulatory mechanism in insects and some other arthropods: a review. J Comp Physiol B 182: 729–740. 10.1007/s00360-012-0651-1 22327195

[34] Grandin LD, Alloy LB, Abramson LY (2006) The social zeitgeber theory, circadian rhythms, and mood disorders: review and evaluation. Clinical Psychology Review 26: 679–694. 10.1016/j.cpr.2006.07.001 16904251

[35] Stanewsky R (2002) Clock mechanisms in *Drosophila*. Cell Tissue Res 309: 11–26. 10.1007/s00441-002-0569-0 12111533

[36] Konopka RJ, Pittendrigh C, Orr D (1989) Reciprocal behaviour associated with altered homeostasis and photosensitivity of Drosophila clock mutants. J Neurogenet 6: 1–10. 2506319 10.3109/01677068909107096

[37] Saunders DS Cymborowski B (2008) Light-induced behavioural effects on the locomotor activity rhythm of the blow fly, *Calliphora cicina* (Diptera: Calliphoridae). Eur J Entomol 105: 585–590.

[38] Payne R, Howard J (1981) Response of an insect photoreceptor: a simple log-normal model. Nature 5805: 416–416.

[39] Wernet MF, Perry MW, Desplan C (2015) The evolutionary diversity of insect retinal mosaics: common design principles and emerging molecular logic. Trends Genet 31: 316–328. 10.1016/j.tig.2015.04.006 26025917 PMC4458154

[40] Böll S, Herrmann JV (2004) A long-term study on the population dynamics of the grape leafhopper (*Empoasca vitis*) and antagonistic mymarid species. J Pest Sci 77: 33–42.

[41] Lessio F, Tedeschi R, Pajoro M, Alma A (2009) Seasonal progression of sex ratio and phytoplasma infection in *Scaphoideus titanus* Ball (Hemiptera Cicadellidae). B Entomol Res 99: 377–383. 10.1017/S0007485308006457 19063761

[42] Briscoe AD, Chittka L (2001) The evolution of color vision in insects. Annu Rev Entomol 46: 471–510. 10.1146/annurev.ento.46.1.471 11112177

[43] Bernard GD, Remington CL (1991) Color vision in Lycaena butterflies: Spectral tuning of receptor arrays in relation to behavioral ecology. Proc Natl Acad Sci USA 88: 2783–2787. 2011588 10.1073/pnas.88.7.2783 PMC51323

[44] Arikawa K, Wakakuwa M, Qiu XD (2005) Sexual dimorphism of short-wavelength photoreceptors in the small white butterfly, Pieris rapae crucivora. J Neurosca 25: 5935–5942. 10.1523/JNEUROSCI.1364-05.2005 PMC6724796 15976082

[45] Hibrant M, Almudi I, Leite DJ, Kuncheria L, Posnien N, Nunes MDS, et al (2014) Sexual dimorphism and natural variation within and among species in the *Drosophila* retinal mosaic. BMC Evol Biol 14: 1–13. 25424626 10.1186/s12862-014-0240-x PMC4268811

[46] De J, Varma V, Saha S, Sheeba V, Sharma VK (2013) Significance of activity peaks in fruit flies, *Drosophila melanogaster*, under seminatural conditions. Proc Natl Acad Sci USA 110: 8984–8989. 10.1073/pnas.1220960110 23671102 PMC3670394

[47] Vanin S, Bhutani S, Montelli S, Menegazzi P, Green EW, Pegoraro M, et al (2012) Unexpected features of *Drosophila* circadian behavioural rhythms under natural conditions. Nature 484: 371–375. 10.1038/nature10991 22495312

[48] Tang ZY, Tang XJ (2010) Effects and forecast of temperature on occurrence of *Empoasca pirisuga* Matumura. J Anhui Agric Sci 38: 3523–3524.

[49] Dietrich C (2005) Keys to the families of Cicadomorpha and subfamilies and tribes of Cicadellidae (Hemiptera: Auchenorrhyncha). Florida Entomologist 88: 502–517.

[50] Heong K, Aquino G, Barrion A (1992) Population dynamics of plant-and leafhoppers and their natural enemies in rice ecosystems in the Philippines. Crop Prot 11: 371–379.

[51] Saeed R, Razaq M, Hardy IC (2015) The importance of alternative host plants as reservoirs of the cotton leafhopper, *Amrasca devastans*, and its natural enemies. J Pest Sci 88: 517–531. 10.1002/ps.4146 26436945

[52] Merckx T, Slade EM (2014). Macro-moth families differ in their attraction to light: implications for light-trap monitoring programmes. Insect Conserv Diver 7: 453–461.

[53] Rai AK, Khan MA (2002) Light trap catch of rice insect pest, *Nephotettix virescens* (Distant) and its relation with climatic factors. Ann Plant Prot Sci 10: 17–22.

[54] Bartlett BR (1968) Outbreaks of two-spotted spider-mites and cotton aphids following pesticide treatment. I. Pest stimulation vs. natural enemy destruction as the cause of outbreaks. J Econ Entomol 61: 297–303.

[55] Chaplin-Kramer R, O’ Rourke ME, Blitzer EJ, Kremen C (2011) A meta-analysis of crop pest and natural enemy response to landscape complexity. Ecol Lett 14: 922–932. 10.1111/j.1461-0248.2011.01642.x 21707902

