# Supplementary figures and images for: Adult Tea Green Leafhoppers, Empoasca onukii (Matsuda), Change Behaviors under Varying Light Conditions
Source: PLoS One. 2017 Jan 19;12(1):e0168439. doi: 10.1371/journal.pone.0168439 (PMC5245864; doi:10.1371/journal.pone.0168439)

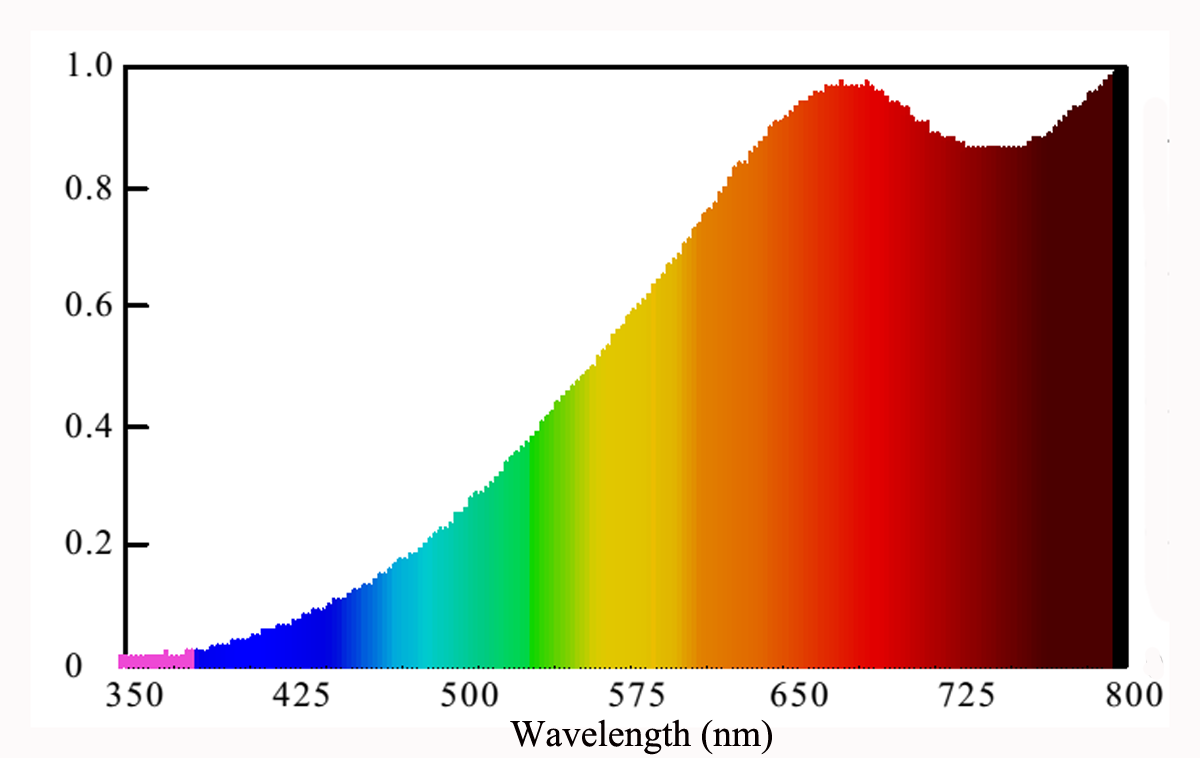

Supplement: S1 Fig — (TIF) [file pone.0168439.s001.tif]

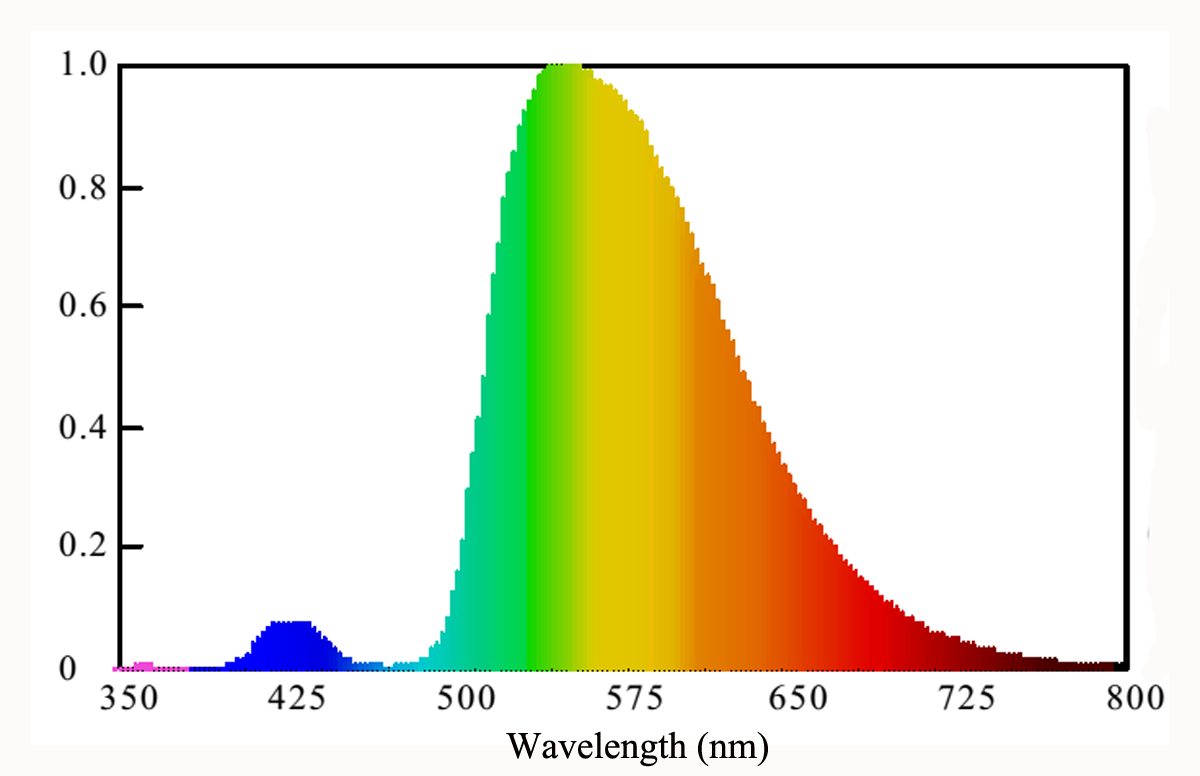

Supplement: S2 Fig — (TIF) [file pone.0168439.s002.tif]

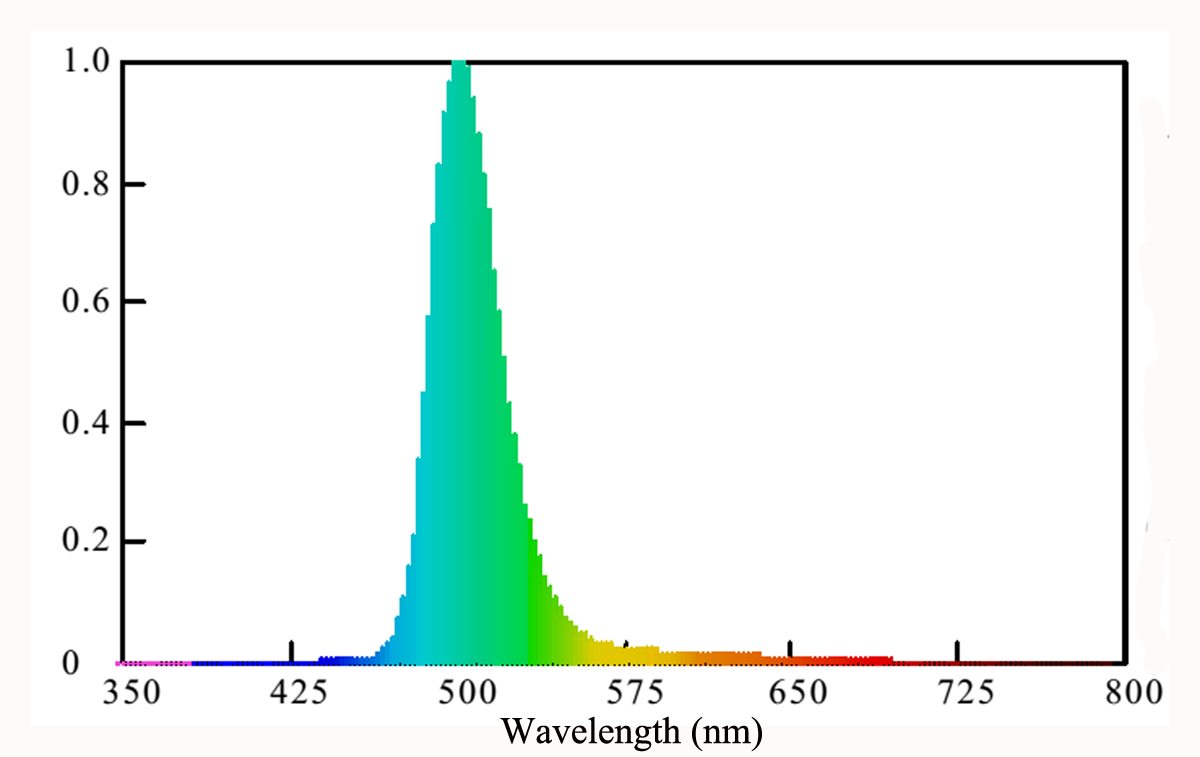

Supplement: S3 Fig — (TIF) [file pone.0168439.s003.tif]

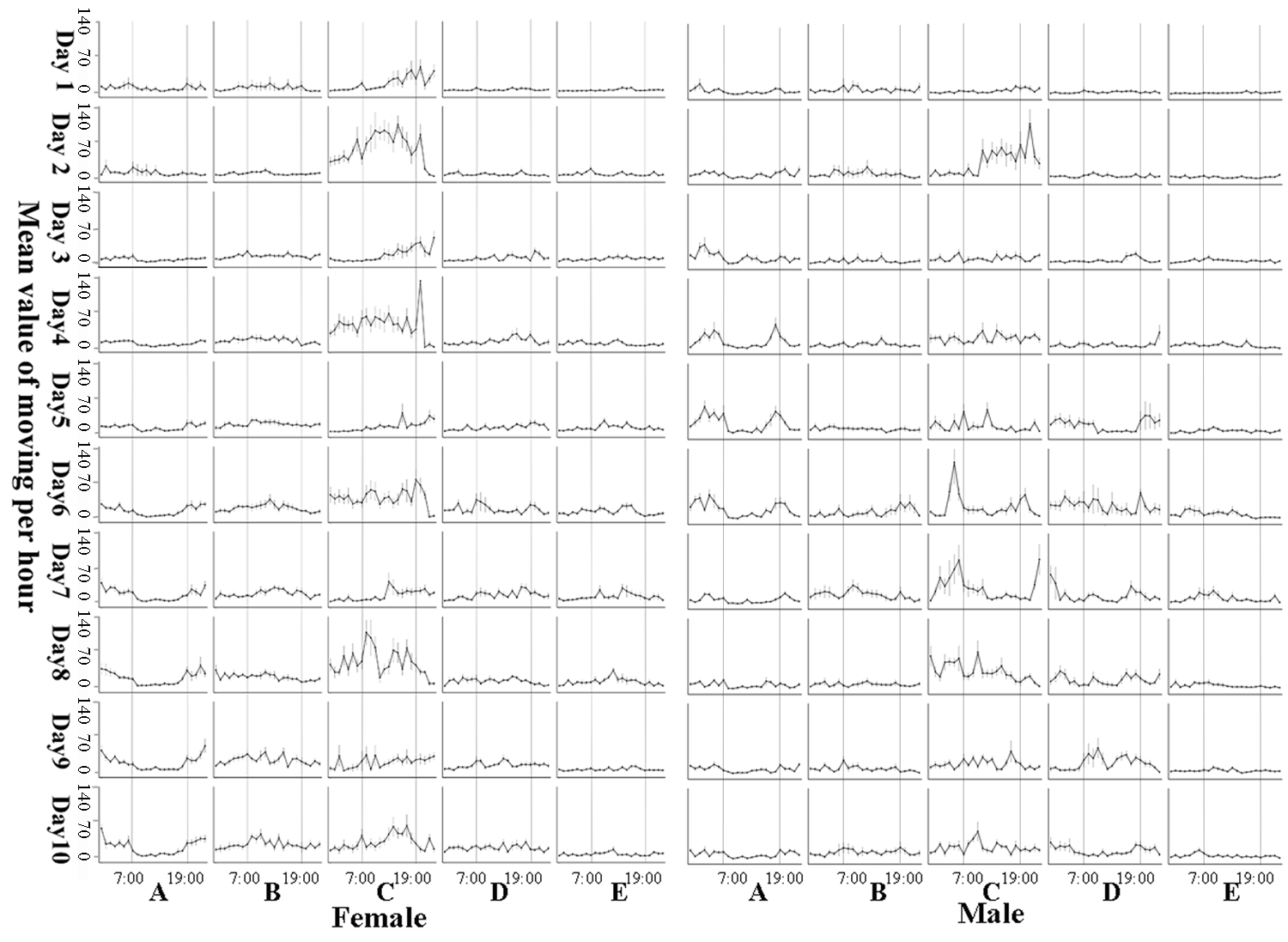

Supplement: S4 Fig — Locomotion events (with standard errors) at each hour of the day for the 10 days of experimentation. For each hour, the mean values of locomotion behaviour of 10 tested leafhoppers in each treatment are shown by line charts with standard errors. Every small chart got the same axis values. Treatments: A) Period 1 with light of quartz lamp, Period 2 with darkness; B) continuous illumination by quartz lamp; C) continuous darkness; D) Period 1 with light of quartz lamp, Period 2 with yellow light (LED); E) Period 1 with light of quartz lamp, Period 2 with green light (LED). Each day (24H) was divided as Period 1(7:00–19:00) and Period 2(19:00–7:00). (TIF) [file pone.0168439.s004.tif]

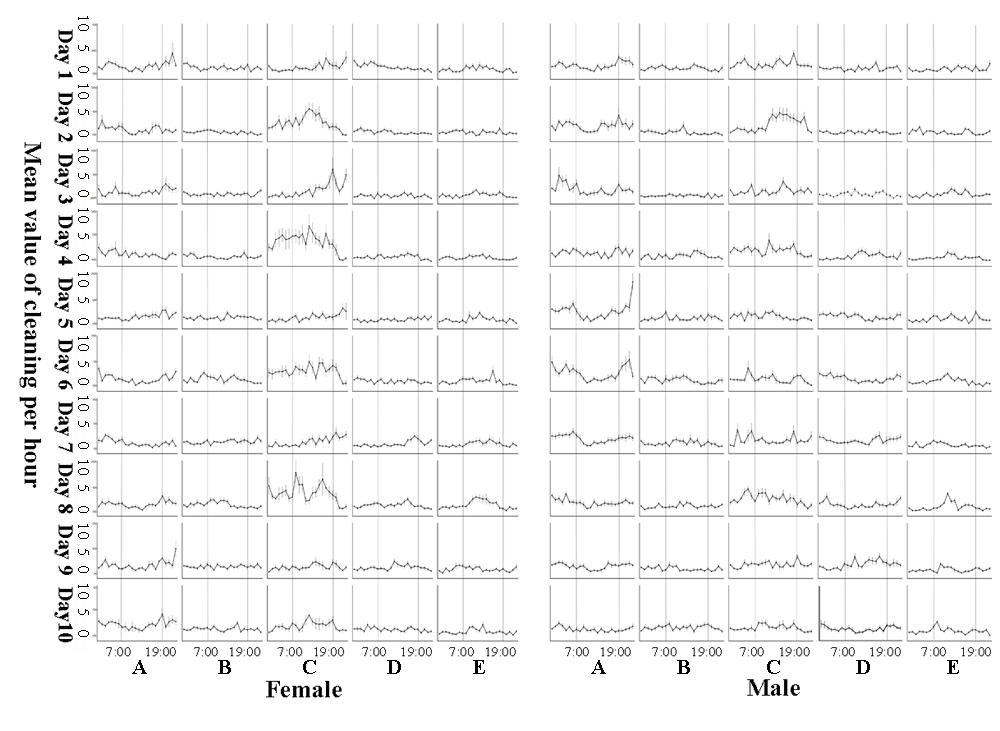

Supplement: S5 Fig — Cleaning events (with standard errors) at each hour of the day for the 10 days of experimentation. For each hour, the mean values of cleaning behaviour of 10 tested leafhoppers in each treatment are shown by line charts with standard errors. Every small chart got the same axis values. Treatments: A) Period 1 with light of quartz lamp, Period 2 with darkness; B) continuous illumination by quartz lamp; C) continuous darkness; D) Period 1 with light of quartz lamp, Period 2 with yellow light (LED); E) Period 1 with light of quartz lamp, Period 2 with green light (LED). Each day (24H) was divided as Period 1(7:00–19:00) and Period 2(19:00–7:00). (TIF) [file pone.0168439.s005.tif]

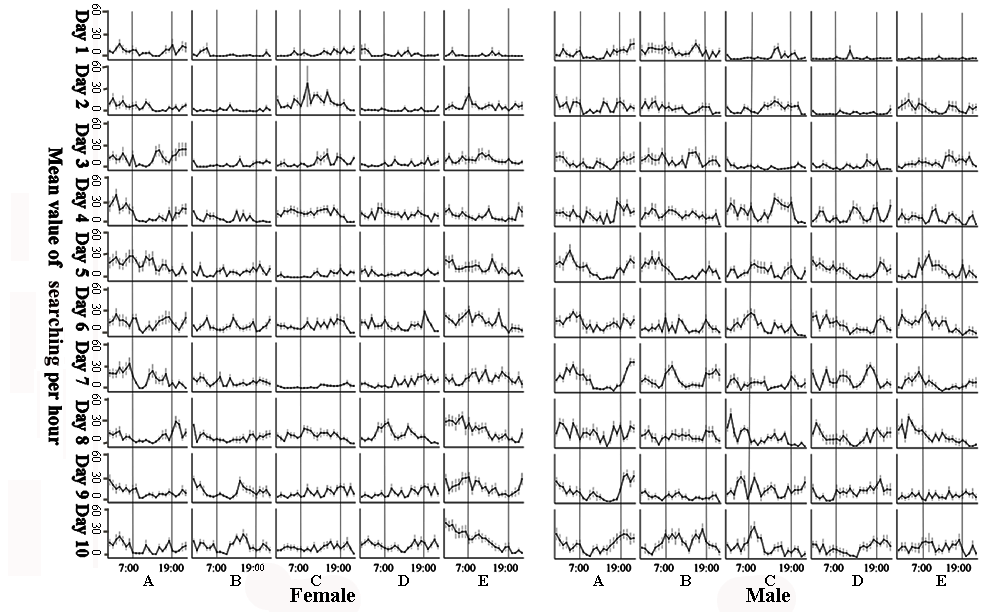

Supplement: S6 Fig — Searching duration (with standard errors) at each hour of the day for the 10 days of experimentation. For each hour, the mean values of searching behaviour of 10 tested leafhoppers in each treatment are shown by line charts with standard errors. Every small chart got the same axis values. Treatments: A) Period 1 with light of quartz lamp, Period 2 with darkness; B) continuous illumination by quartz lamp; C) continuous darkness; D) Period 1 with light of quartz lamp, Period 2 with yellow light (LED); E) Period 1 with light of quartz lamp, Period 2 with green light (LED). Each day (24H) was divided as Period 1(7:00–19:00) and Period 2(19:00–7:00). (TIF) [file pone.0168439.s006.tif]
